# Random topology organization and decreased visual processing of internet addiction: Evidence from a minimum spanning tree analysis

**DOI:** 10.1002/brb3.1218

**Published:** 2019-01-31

**Authors:** Hongxia Wang, Yan Sun, Jiaojiao Lv, Siyu Bo

**Affiliations:** ^1^ School of Psychology Liaoning Normal University Da Lian China

**Keywords:** EEG, functional connectivity, internet addiction, minimum spanning tree, phase lag index

## Abstract

**Objectives:**

Internet addiction (IA) has been associated with widespread brain alterations. Functional connectivity (FC) and network analysis results related to IA are inconsistent between studies, and how network hubs change is not known. The aim of this study was to evaluate functional and topological networks using an unbiased minimum spanning tree (MST) analysis on electroencephalography (EEG) data in IA and healthy control (HC) college students.

**Methods:**

In this study, Young's internet addiction test was used as an IA severity measure. EEG recordings were obtained in IA (*n* = 30) and HC participants (*n* = 30), matched for age and sex, during rest. The phase lag index (PLI) and MST were applied to analyze FC and network topology. We expected to obtain evidence of underlying alterations in functional and topological networks related to IA.

**Results:**

IA participants showed higher delta FC between left‐side frontal and parieto‐occipital areas compared to the HC group (*p* < 0.001), global MST measures revealed a more star‐like network in IA participants in the upper alpha and beta bands, and the occipital brain region was relatively less important in the IA relative to the HC group in the lower band. The correlation results were consistent with the MST results: higher IA severity correlated with higher Max degree and kappa, and lower eccentricity and diameter.

**Conclusions:**

Functional networks of the IA group were characterized by increased FC, a more random organization, and a decrease of relative functional importance of the visual processing area. Taken together, these alterations can help us understand the influence of IA to brain mechanism.

## 1 INTRODUCTION

The consequences of the popularity of the internet are both beneficial and disadvantageous, while excessive use of the internet may lead to internet addiction (IA). The University of Pittsburgh's Psychology Professor Young defined the IA as the excessive or uncontrolled use of the internet with negative consequences to psychological, social, and/or work functioning aspects (Dong, Lin, & Potenza, 2015; Young, 1998). Studies have shown that IA is widespread throughout the world (Block, 2008). In addition, IA can lead to sleeping, mood, academic problems, and other physical and mental health problems (Canan et al., 2013; Zainudin, Din, & Othman, 2013).

In recent years, with the advancement of neurological imaging detection techniques, structural and functional alterations of multiple brain regions have been found in IA. Structurally, IA alters gray matter density, gray matter volume, fractional anisotropy, and cortical thickness compared to controls (Han, Lyoo, & Renshaw, 2012; Hong, Kim et al., 2013; Lin et al., 2012; Yuan et al., 2013, 2011; Zhou et al., 2011). Lee et al. (2017) found evidence that IA resulted in structural abnormalities which may be associated with functional impairments. In resting‐state function, IA has been associated with significant functional changes in corticostriatal circuits (Hong, Zalesky et al., 2013), regions located in the frontal, occipital, and parietal lobes (Wee et al., 2014), the visual information‐processing circuits, and the prefrontal areas (Koo et al., 2008; Wen & Hsieh, 2016). Wang et al. (2017) found altered default mode, frontoparietal, and salience networks in adolescents with IA. Collectively, the altered brain regions related to IA are often widely distributed, and the conclusions of brain connectivity have been inconsistent or even contradictory. For instance, some studies found that adolescents with IA exhibited increased coherence compared to HC participants regardless of psychological features (e.g., depression, anxiety, and impulsivity) (Kwan & Choi, 2015; Park et al., 2017), while others found that IA appears to result from reduced connectivity (Hong, Zalesky et al., 2013; Wee et al., 2014). One of the aims of this study was to explore the characteristics of brain connectivity in IA.

The human brain is a highly organized and complex network, a large‐scale structural and functional integration network. The brain has a small‐world architecture, combining such optimal properties as the high clustering of an ordered network and the short path length of a random network (Boersma et al., 2011), to ensure that it can quickly deal with external stimuli to achieve cognitive function (Bullmore & Sporns, 2012). The development of graph theory provides a perfect tool for neurological analysis (Rubinov & Sporns, 2010). Graph theory can fully characterize the structure and function of brain networks, and provide the basic properties of neural propagation structures and dynamic organization (Bullmore & Sporns, 2012). More and more research has used graph theory to study IA. Zhai et al. (2017) used diffusion tensor imaging (DTI) tractography to thoroughly characterize topological property changes of the white matter (WM) network at the circuit level in patients with internet gaming disorder (IGD). The IGD group showed decreased global efficiency, decreased local efficiency, and increased shortest path length compared to controls, further demonstrating that IGD involves a less integrated network organization. Hong, Zalesky et al. (2013) used functional magnetic resonance imaging (fMRI) technology to explore the network topology of internet addicts, and no group difference was observed in network topological measures, including the clustering coefficient, characteristic path length, or the smallworldness ratio. Lee et al. (2017) constructed a structural brain network from DTI data and found that the subjects with IA showed increased regional efficiency in the bilateral orbitofrontal cortex and a decrease in the right middle cingulate and middle temporal gyri, whereas the global properties did not show significant changes. This is consistent with Wee et al. (2014), who also confirmed that although significant alterations were observed for regional nodal metrics, there was no difference in global network topology between IA and healthy groups.

According to the above results, different scholars have reached different, even conflicting, conclusions. This may be due to the different choices of threshold T in the process of constructing traditional brain networks; smaller T may result in false or noisy connections in the network, while larger T may discard some links that contain important information. The network properties in traditional networks are sensitive to network sparseness (van Diessen et al., 2015). To improve the accuracy of network construction and the feasibility of comparative analysis between different networks, the minimum spanning tree (MST) method was introduced into brain network analysis (Tewarie, van Dellen, Hillebrand, & Stam, 2015; van Diessen et al., 2015). MST is the only acyclic subgraph containing the strongest connection in the original undirected weighted network. Considering that the exchange of information in the original network is always based on the most efficient path, the MST can be considered the backbone of the functional brain network (van Diessen et al., 2015). The number of edges in the MST is equal to *N *– 1 (*N* represents the number of nodes in the MST), which guarantees that when the number of nodes in two compared network is the same, they will also have the same number of edges. In this case, what compares is the difference in purely topological attributes. MST avoids methodological biases and is particularly suitable for comparison of brain networks (Tewarie et al., 2014). MST is effective to explore the brain mechanisms of various populations, such as epileptic (Lee, Kim, & Jung, 2006; van Dellen et al., 2014; van Diessen, Otte, Stam, Braun, & Jansen, 2016), depressive (Fraga et al., 2016), dyslexic (Fraga et al., 2016), and healthy subjects (Boersma et al., 2013; Demuru, Fara, & Fraschini, 2013). As far as we know, there is still no research on IA based on MST.

Although structural and functional studies have discovered some altered brain regions related to IA, there is still limited evidence based on resting‐state EEG data about whether IA can cause changes in the overall brain properties and whether the hubs that play important roles in functional networks change. Therefore, the goal of the current study was to examine global functional network connectivity and organization and detect the hubs between IA and controls in resting‐state EEG data. Studying the dynamics of spontaneous (independent‐task) activities in the brain provides us with meaningful information on how the different brain regions communicate and the functional brain network infrastructure (Fraga et al., 2016; Li et al., 2017gies, such as fMRI (Khanna, Pascual‐Leone, Michel, & Farzan, 2015). In addition, although traditional graph theory analysis is helpful for understanding brain mechanisms, it still has the limitation of a lack of standard methods (van Diessen et al., 2016). Thus, we attempted to introduce a recent development in graph theory as applies to MST analysis to explore the changes in brain mechanisms related to IA.

## 2 MATERIALS AND METHODS

### 2.1 Participants

Participants in this study were all college students of Liaoning Normal University evaluated by internet addiction test (IAT), including 30 IA (7 males, IAT range was 50–75) and 30 matched healthy control (HC) participants (6 males, IAT range was 20–49). The exclusion criteria included (a) symptoms of mental illness, such as depression, anxiety or attention‐deficit/hyperactivity disorder (ADHD); (b) a history of alcohol, nicotine or drug use; (c) pregnant or menstruating women; and (d) a history of brain injury. All participants were native Chinese speakers, and they had normal or corrected‐to‐normal vision. Specific participant information is shown in Table 1. The study was conducted in accordance with the recommendations of the Liaoning Normal University Ethics Committee, and all participants had signed the informed consent. Participants were required to ensure sufficient sleep during the night before they did the experiment and avoided contact with the internet the night before the experiment.

**Table 1:** Statistics of basic information of the participants

Group	Age $\bar{x}\pm s$	Sex (Male/Female)	Profitable hand (Right/Left)	IAT $\bar{x}\pm s$
IA	21.0 ± 2.13	7/23 (30)	30/0	59.0 ± 7.88
HC	20.5 ± 1.59	6/24 (30)	30/0	34.4 ± 8.53
*p*	0.241^a^	0.719^b^	—	<0.0001^a^

Note

“a” and “b” denote two‐sample *t* test and Pearson chi‐square test, respectively.

### 2.2 Equipment and Procedure

#### 2.2.1 Internet Addiction Test

The IAT was compiled by Young (1998) at the University of Pittsburgh. The scale is self‐reported and contains 20 items. The title options for rarely, occasionally, sometimes, often and always are scored as 1, 2, 3, 4, and 5 points, respectively. The total score is 20–100, with higher scores representing higher levels of IA: 20–49 for normal users, 50–79 for excessive internet addicts, and 80–100 for severe internet addicts. This measure has demonstrated good reliability and validity in Chinese (Zhou, Li, Xian, Wang, & Zhao, 2017).

#### 2.2.2 Experimental Procedure

The experiment was run in a well‐shielded, soundproofed room where participants comfortably sat in an armchair. We explicitly clarified the experimental requirements to the participants prior to the start of the experiment. The task‐independent resting‐state EEG signals of each participant were collected. During the six‐minute EEG signal acquisition process, participants were asked to close their eyes, relax, avoid large head movements, and not think about anything but had to stay awake and not sleep. EEG recordings of all participants were monitored throughout to ensure that they followed the instructions and did not show signs of drowsiness.

### 2.3 EEG recording and signal processing

A digital EEG recording system produced by the Brain‐Product company (German) was adopted. The 64‐channel electrode cap was complied with the 10–20 International System. The electrode points and their corresponding numbers were as follows: 1–10: FP1, FP2, F3, F4, C3, C4, P3, P4, O1, O2; 11–20: F7, F8, T7, T8, P7, P8, Fz, Cz, Pz, IO; 21–30: FC1, FC2, CP1, CP2, FC5, FC6, CP5, CP6, FT9, FT10; 31–40: TP9, TP10, F1, F2, C1, C2, P1, P2, AF3, AF4; 41–50: FC3, FC4, CP3, CP4, PO3, PO4, F5, F6, C5, C6; 51–60: P5, P6, AF7, AF8, FT7, FT8, TP7, TP8, PO7, PO8; and 61–64: FPz, CPz, POz, Oz. Both the vertical and horizontal channels of the EOG were recorded simultaneously to monitor the eye movements and blinks. The unipolar reference region was linked at the right and left earlobes, and the ground electrode was located at the AFz (A‐Ear lobe, F‐Frontal lobe, z‐zero, referring to an electrode placed on the midline). The sampling frequency was 500 Hz, and the electrode impedance was less than 10 KΩ.

Offline data of 30 IAs and 30 HCs were analyzed by Brain Vision Analyzer 2 software. First, data were re‐referenced to the mastoid channels, then were low‐pass‐filtered using a cut‐off frequency of 256 Hz and bandpass‐filtered between 0.5 and 50 Hz to exclude very low‐frequency artifacts and line noise. Data portions contaminated by eye movements, electromyography, or any other nonphysiological artifacts were corrected using the independent component analysis algorithm (Jung et al., 2001; Makeig, Jung, Bell, Ghahremani, & Sejnowski, 1997). Then, the preprocessed 6‐min continuous EEG data were segmented into dozens of epochs, with an epoch length of 2000 ms. EEG epochs contaminated by strong muscle artifacts or with amplitude values exceeding ±150 μV at any electrode were manually rejected. Finally, a minimum of 80 epochs were considered sufficient for further analysis. The artifact‐free epochs were exported to ASCII files and imported in Brainwave v0.9.151.7.2. (developed by Cornelis Jan Stam; freely available at http://home.kpn.nl/stam7883/brainwave.html).

### 2.4 Phase lag index (PLI)

When the EEG functional brain network was constructed, electrode channels were generally defined as nodes. The definition of edges was mainly to measure the correlation between time series of different channels. The PLI method was selected in current study. Its biggest advantage is that it only depends on the phase difference between the two signals and is not affected by the volume conductor effect (Stam, Nolte, & Daffertshofer, 2007). The PLI is obtained from the time series of phase differences Δφ(*t_k_*), *k *= 1…*N* by means of:

$$\text{PLI}=|<\text{sign}[\sin\left(\Delta \varphi \left(t_{k}\right)\right)]>|$$

Here, sign is the signum function. The PLI quantifies the asymmetry of the relative phase distribution; that is, the likelihood that the phase difference Δ*φ* will be in the interval –π < Δ*φ* < 0 is different from the likelihood that it will be in the interval 0 < Δ*φ* < π. This means that there is a consistent non‐zero phase difference ('lag') between the two time series. If there is no coupling or if the median phase difference is equal to or centered on the value of 0 mod *π*, then the expected distribution is symmetrical (Fraga et al., 2016). The PLI ranges between 0 and 1; the higher the PLI of the two nodes, the stronger the correlation between the two brain regions. The PLI is effective at detecting real changes in functional networks (Fraga et al., 2016; van Dellen et al., 2014; van Diessen et al., 2016). We constructed the undirected and weighted brain networks for each participant by calculating the PLI values between 64 electrodes for each band (delta: 0.5–4 Hz; theta: 4–8 Hz; alpha1: 8–10 Hz; alpha2: 10–13 Hz; beta: 13–25 Hz; gamma: 25–49 Hz).

### 2.5 Minimum spanning tree (MST)

We constructed the MST, which is the core part of the network with the largest total weight. It connects all the nodes in the network and does not constitute a loop. Extreme topologies of MST are, on one hand, a star‐like or centralized organization and, on the other hand, a decentralized line‐like tree. The star‐like and line‐like organization may be translations of, respectively, random and ordered networks (Boersma et al., 2013). Examples of traditional networks and tree topologies are presented in Figure S1. This study used the Kruskal algorithm (Kruskal, 1956) to build the MST, which contained 64 nodes. The construction process was as follows: First, all linked weights in the PLI matrix were sorted in descending order, and then the links were added in order of weight, starting with the largest. During this process, we discarded the link if the added link constituted a loop. The Kruskal algorithm terminated until all nodes were included.

To investigate the global topological organization of IA, some MST network characteristics were quantified. Degree centrality (Deg) is the number of edges connected to a node. Betweenness centrality (BC) is a measure of the node's “hub” within the network. It is defined as the normalized fraction of all shortest paths connecting two nodes that pass through the particular node. The Deg and BC measures were calculated for each node separately, and the maximum values within each MST were included in the statistical analyses as global characteristics of the MST (named MaxDeg and MaxBC, respectively). Eccentricity (Ecc) is the longest shortest path from a particular node to any other node in the network. Based on the definition of Ecc, diameter (Diam) is the largest distance between any two nodes within the MST; smaller diameter values denote better network cohesion. Leaf fraction (Leaf) is the number of nodes with only one connected edge divided by the total number of nodes in the MST. Tree hierarchy (Th) assesses how hierarchical a given network is. Th ranges from 0 (indicating a line‐like topology) to 1; for star‐like topology, Th approaches 0.5. The higher the Th is, the better the tradeoff between integration and segregation is in an MST. Kappa (K) is mainly related to the synchronization level of tree nodes. Finally, degree correlation (*R*) is computed through the Pearson correlation coefficient of the degrees of pair of vertices connected by an edge. For a detailed description of the various metrics, please refer to Fraga et al. (2016).

In addition, Deg and BC can be used as hub indicators. Nodes with the highest BC or Deg values were characterized as critical nodes (hubs) and were used to determine the information flow within the network.

### 2.6 Statistical Analysis

One‐way ANCOVA was used for group comparisons of PLI averages and global MST measures, including the age, sex, and power of each frequency band as covariates. The global PLI and MST network characteristics were averaged across epochs, separately for each participant and frequency band. Bonferroni correction for multiple comparisons was applied to *p *values for each frequency band. Additionally, the two‐sample *t* test was applied to explore the regions with significant differences between groups based on Deg and BC. We also detected the hub locations of the IA and HC groups based on the highest BC and Deg values. Finally, to examine the relationship between the FC, topological measures, and IA severity, we computed Pearson's correlation coefficient between global or local significance measures and participants’ IAT values.

## 3 RESULTS

### 3.1 FC and global MST

**Figure 1:**
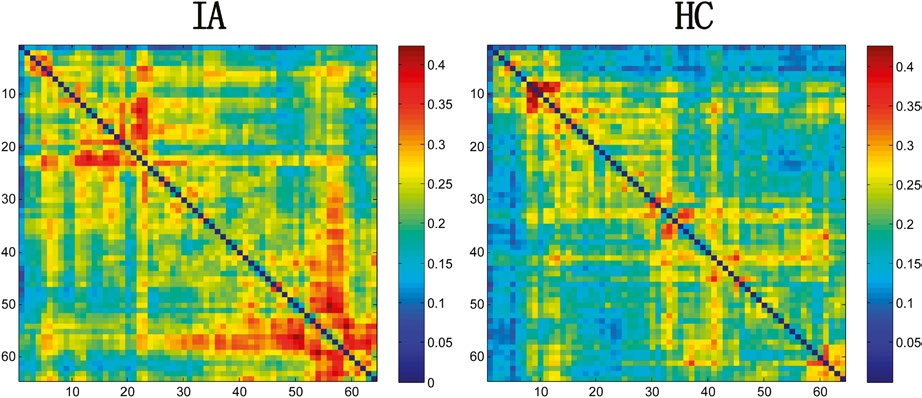
The PLI of the IA and HC groups in the delta band. The size of the PLI matrix was 64*64. In the matrix map, each chromatic point represents the synchronization of two corresponding channels. The horizontal and vertical axes denote 64 channels. The right color bar represents the connection strength, from blue to red indicates increasing connection strength

**Figure 2:**
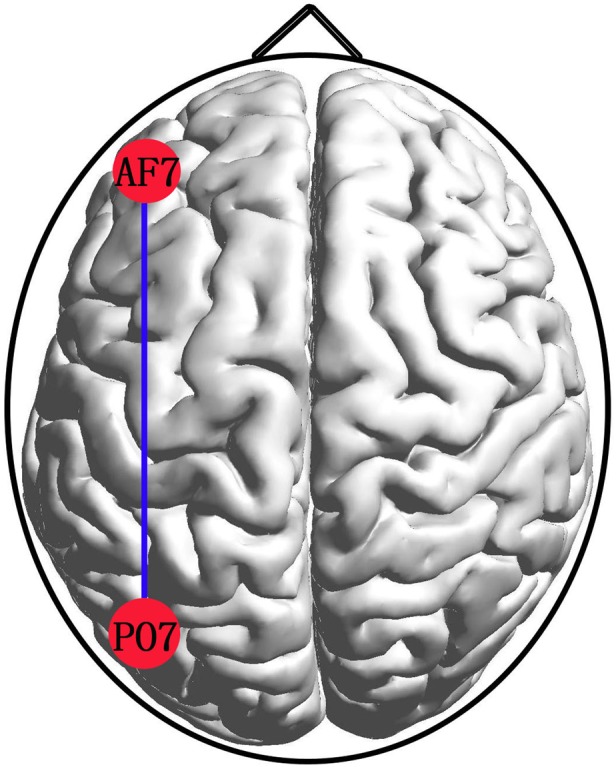
EEG network shows significantly increased synchronization in the IA group compared to the HC group in delta band (*p* < 0.001). No significant changes were observed in other regions and bands

The results of the ANCOVAs performed on the PLI, MaxDeg, Ecc, MaxBC, K, R, Diam, Leaf, and Th in each frequency band are presented in Table 2 (Figure S2). All PLI values from the connectivity matrix were averaged separately for each participant, and the ANCOVAs yielded a significant delta difference in FC between groups (*F* = 4.580, *p* = 0.033, η^2^ = 0.013). The delta square PLI matrix is presented in Figure 1 for illustration purposes, and the connectivity between the left frontal (AF7) and left parietooccipital (PO7) was significantly increased in the IA group (0.368 ± 0.301) over the HC group (0.132 ± 0.190) (*p* < 0.001) (Figure 2). No significant difference was detected in other bands.

**Table 2:** Results of global PLI and MST measures in delta, alpha2, and beta frequency bands

	IA (*N* = 30)		HC (*N* = 30)		*F*	*P*	η^2^
	M	*SD*	M	*SD*			
Delta							
PLI	**0.231**	**0.082**	**0.202**	**0.071**	**4.580**	**0.033↑**	**0.013**
Max degree	0.497	0.227	0.490	0.201	0.015	n.s.	0.000
Eccentricity	0.105	0.041	0.098	0.033	0.519	n.s.	0.002
Max BC	0.833	0.107	0.822	0.098	0.168	n.s.	0.000
Kappa	12.478	6.730	12.043	6.179	0.040	n.s.	0.000
*R*	−0.608	0.128	−0.620	0.133	0.044	n.s.	0.000
Diameter	0.131	0.054	0.119	0.043	0.875	n.s.	0.003
Leaf	0.849	0.100	0.859	0.098	0.342	n.s.	0.001
Th	0.513	0.058	0.527	0.071	1.387	n.s.	0.004
Alpha2							
PLI	0.028	0.028	0.029	0.033	0.007	n.s.	0.000
Max degree	**0.873**	**0.213**	**0.768**	**0.213**	**3.859**	**0.050↑**	**0.011**
Eccentricity	**0.056**	**0.039**	**0.085**	**0.039**	**6.608**	**0.011↓**	**0.019**
Max BC	0.961	0.084	0.925	0.094	2.120	n.s.	0.006
Kappa	**26.467**	**8.301**	**21.402**	**8.743**	**6.243**	**0.013↑**	**0.018**
*R*	**−0.775**	**0.292**	**−0.615**	**0.272**	**6.767**	**0.010↓**	**0.019**
Diameter	**0.064**	**0.009**	**0.094**	**0.009**	**5.941**	**0.015↓**	**0.017**
Leaf	*0.957*	*0.086*	*0.919*	*0.078*	*3.563*	*0.060*	*0.010*
Th	0.498	0.025	0.498	0.026	0.001	n.s.	0.000
Beta							
PLI	0.048	0.037	0.060	0.059	1.057	n.s.	0.003
Max degree	**0.845**	**0.185**	**0.710**	**0.219**	**6.773**	**0.010↑**	**0.019**
Eccentricity	0.059	0.026	0.075	0.037	3.168	n.s.	0.009
Max BC	**0.950**	**0.086**	**0.902**	**0.107**	**4.152**	**0.042↑**	**0.012**
Kappa	**25.014**	**7.271**	**19.480**	**8.141**	**7.868**	**0.005** a **↑**	**0.022**
*R*	**−0.832**	**0.147**	**−0.698**	**0.184**	**4.988**	**0.026↓**	**0.014**
Diameter	**0.066**	**0.034**	**0.090**	**0.053**	**3.938**	**0.048↓**	**0.011**
Leaf	*0.961*	*0.041*	*0.928*	*0.073*	*2.922*	*0.088*	*0.008*
Th	0.509	0.044	0.520	0.063	0.967	n.s.	0.003

Note

Bold text represents significant results (*p < 0.05*), italic text represents results at trend level. MST: minimum spanning tree; PLI: phase lag index; BC: betweenness centrality; R: degree correlation; Th: tree hierarchy.

↑ = IAs > HCs, ↓ = IAs<HCs, n.s. = nonsignificant.

a *p < *0.006 (0.05/9), significant after Bonferroni correction.

MST analysis yielded significant effects between groups in the upper alpha and beta bands (see Table 2). Deg and BC are related to the importance of a node within the network. The MaxDeg was significantly higher in the IA group relative to the HC group in both the upper alpha and beta bands, *F* = 3.859, *p* = 0.050, η^2^ = 0.011, and *F* = 6.773, *p* = 0.010, η^2^ = 0.019, respectively. MaxBC was only significantly higher in the IA group in the beta band, *F* = 4.152, *p = *0.042, η^2^ = 0.012*. *Ecc, another measure of relative nodal importance that is low if this node is central in the tree, was significant in the upper alpha band, *F* = 6.608, *p* = 0.011, η^2^ = 0.019. Diam, reflecting the efficiency of communication between the nodes, was significantly lower in the IA group in the upper alpha and beta bands, *F* = 5.941, *p* = 0.015, η^2^ = 0.017, and *F* = 3.938, *p* = 0.048, η^2^ = 0.011, respectively. K and *R* are mainly related to the synchronization level of tree nodes. The K of the IA group was significantly higher than the HC group in both the upper alpha and beta bands, *F* = 6.243, *p* = 0.013, η^2^ = 0.018, and *F = *7.868, *p = *0.005, η^2^ = 0.022. The R of the IA group was significantly lower than the HC group in both the upper alpha and beta bands, *F* = 6.767, *p = *0.010, η^2^ = 0.019*,* and *F = *4.988, *p = *0.026, η^2^ = 0.014, respectively. Leaf, reflecting the integration of information within the network, fell short of significance in both the upper alpha and beta bands, *F = *3.563, *p = *0.060, η^2^ = 0.011, and *F = *2.922, *p = *0.088, η^2^ = 0.008, respectively, suggesting a trend for higher leaf fraction in the IA group compared to the HC group. Group effects in all other measures and frequency bands were not significant (Table S1 and Figure S2).

### 3.2 Hubs and regional MST properties

**Figure 3:**
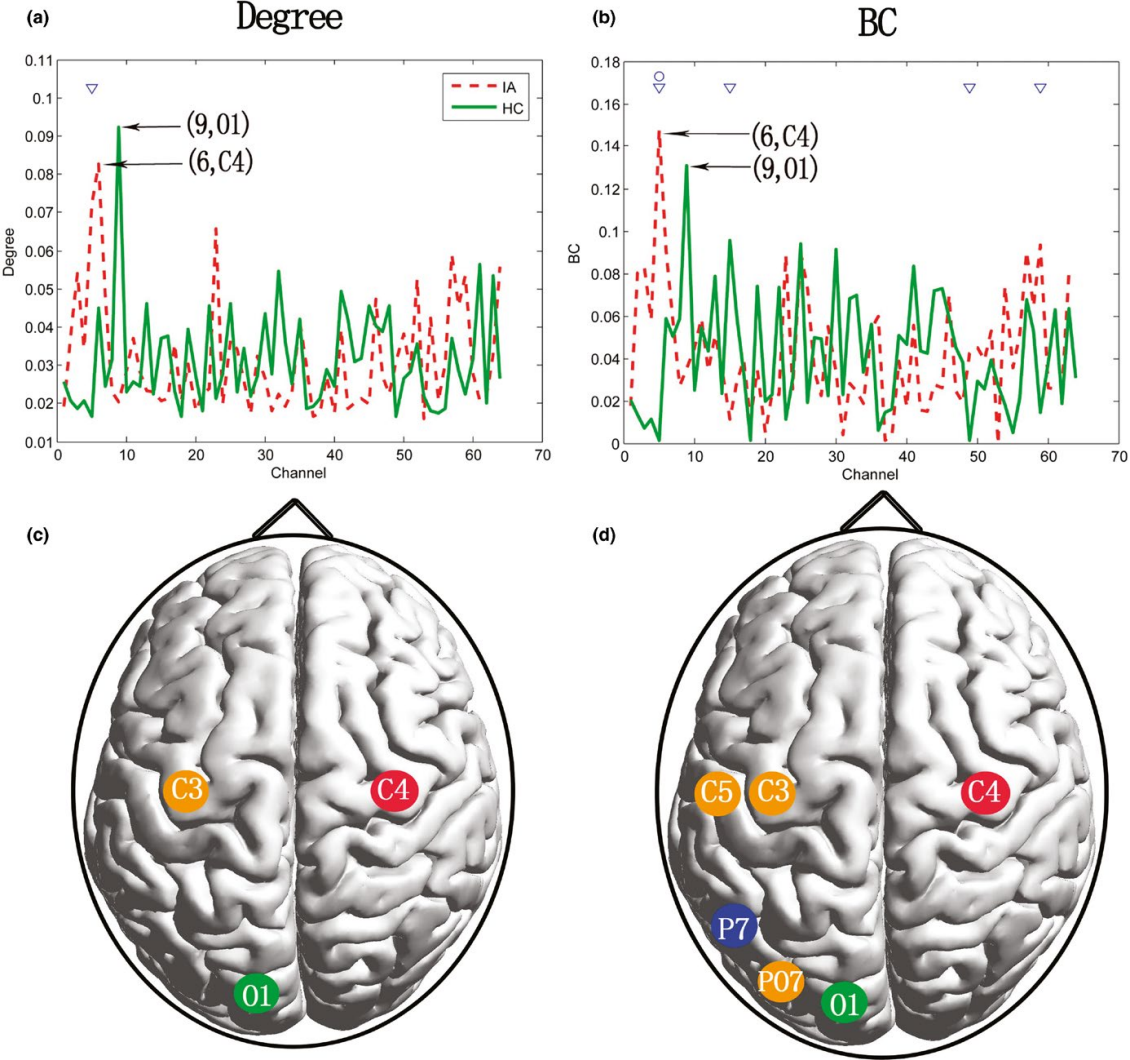
Hub locations and group differences in regional properties based on Deg and BC in delta band. In (a), the *x*‐axis represents 64 channels, *y*‐axis represents degree, the red dotted line and the green solid line represent the IA and HC groups, respectively. The channel pointed by the arrow was the one where the maximum degree lied and marked the name of the channel and the corresponding number. ‘▽’ refers to *p* < 0.05; group difference occurred at electrode point C4 numbered 5. The legend of (b) was consistent with (a) and the y‐axis represents BC and ‘○’ refers to *p* < 0.01; group differences occurred at electrode points C3, P7, C5, PO7 numbered 5, 15, 49, 59 respectively. (c) and (d) show the brain topological position of the hub regions and significant brain regions based on Deg and BC, respectively. The node with green color refers to: MaxDeg/MaxBC node of HC group; red to MaxDeg/MaxBC node of IA group; orange to IAs > HCs; and blue to IAs < HCs

The Deg and BC values of the nodes in the MST were used as an indication of the node importance. A node with MaxDeg or MaxBC can be seen as the “hub” in the network (Pezoulas, Zervakis, Michelogiannis, & Klados, 2017). Thus, we performed a regional analysis based on Deg and BC in delta, alpha2, and beta bands because of the global differences appeared in these bands. We found significant group differences only in the delta band. Hub analysis revealed that the highest BC and Deg values appeared in the left occipital region (O1) in the HC group and in the right central region (C4) in the IA group, (Figure 3). Locally, the IA group showed a significantly higher degree in the left central region (C3) than the HC group. BC was higher in the central (C3, C5) and parietooccipital regions (PO7) and lower in the parietal region (P7) in the IA group than the HC group (Figure 3). Thus, the position of the hub of the IA group presented a back‐to‐center shifting from the occipital to central regions. In addition, the regional difference between groups was mainly located in the left central and parietooccipital regions.

### 3.3 Correlation between global MST measures and IA severity

Pearson correlation was conducted between all significant global and local measures and subjects’ IAT scores. The results showed a significant positive correlation between MaxDeg, K, and IA severity in both the upper alpha (*r = *0.284, *p = *0.028 and *r = *0.318, *p = *0.013, respectively) and beta bands (*r = *0.275*, p = *0.034 and *r = *0.302, *p = *0.019, respectively). There was a significant negative correlation between Ecc, Diam, and IA severity just in the upper alpha band (*r *= −0.310, *p* = 0.016 and *r *= −0.299, *p* = 0.020, respectively) (Table 3). There were no significant correlations between functional connectivity, regional MST measures, other global MST measures, and IA severity.

**Table 3:** Significant correlations between IAT and global MST measures in upper alpha and beta bands

Bands	Measures	IAT (*n* = 60)	
		*r*	*p*
Alpha2	Max degree	0.284	0.028*
	Ecc	−0.310	0.016*
	K	0.318	0.013*
	Diameter	−0.299	0.020*
Beta	Max degree	0.275	0.034*
	K	0.302	0.019*

Note

“*” indicates statistical significant results (*p* < 0.05).

## 4 DISCUSSION

In this study, we aimed to further elucidate functional network alterations in college students with IA as measured with resting‐state EEG combined with a new methodological development in network analysis. We found that a significant increase in the FC between the left frontal (AF7) and left parietooccipital (PO7) lobes in the IA group relative to HCs restricted to the delta band. In addition, college students with IA demonstrated higher MaxDeg and K and lower Ecc, R, and Diam in the upper alpha band, which indicated a shift toward a more centralized star‐like and random network topology compared to the HC group. Comparable results were obtained in the beta band, showing higher MaxDeg, MaxBC, and K and lower R and Diam. Locally, the location of the hub quantified with the BC and Deg of each electrode point in the functional network of IA participants toward the central brain regions was comparable to the hub of the HC group located in the occipital region. The regional difference between groups was mainly located in the left central and parietooccipital regions. In general, our correlation results were consistent with our MST results: higher IA severity was correlated with higher MaxDeg and K and lower Ecc and Diam.

### 4.1 Increased FC between left frontal and parietooccipital regions

Higher FC in IA has previously been reported in studies using different modalities, but the pattern of this FC and the methods vary considerably between studies. In many studies that used lower temporal resolution but high spatial resolution fMRI, an increased FC pattern was found (Du et al., 2017; Han, Kim, Bae, Renshaw, & Anderson, 2017; Hong, Sun, Bae, & Han, 2018). Such a correlation between increased FC and IA may be interpreted as a constructive, adaptive effect of prolonged internet use forming a training effect (Han et al., 2017). Another interpretation, by Wang et al. (2017), was that it was possible that IA was associated with shared disturbances of lower interhemispheric and higher intrahemispheric functional connection. Our result of a significant increase in the FC between the left frontal and left parietooccipital lobes in the IA group relative to HCs also supports this higher intrahemispheric connection. This frontoparietal network connection is implicated in a wide range of cognitively demanding tasks (Wang et al., 2017), the frontal and parietal lobes are both involved in attention networks (Corbetta & Shulman, 2002), and a larger and more bilateral frontoparietal network is activated in a short‐term memory task (Deprez et al., 2013).

Decreased FC correlated with IA is also reported (Dong et al., 2015; Hong, Zalesky et al., 2013). These results indicate that the deceased coherence of brain activity in IA participants may underlie impaired executive function and weakened inhibition control of internet‐using behaviors. In some studies, both a regional increase and a decrease were found (Ding et al., 2013; Wang et al., 2017). Several factors may explain the difference in connectivity patterns related to IA. First, differences between IA participants in various studies have been observed. For example, different criteria for IA (Chen Internet Addiction Scale or Young Internet Addiction Test) and different cut‐offs for the severity of IA have been used. Second, whether IA was accompanied by other psychological syndromes (e.g., depression, ADHD), which has a great influence on the results (Han et al., 2017). Third, the applied technologies and connectivity methods. EEG and fMRI are sensitive to, respectively, fast and slow time scales, and fMRI provides an indirect measure of neuronal activity, unlike EEG (Janssen et al., 2017). Functional networks reconstructed on the basis of fMRI may therefore more closely reflect gross underlying structural networks (Honey, Kötter, Breakspear, & Sporns, 2007), and EEG studies measure the consistency of synchronization activities between time‐series signals.

### 4.2 Centralized and star‐like network topology from global MST attributes

The MST method is an unbiased estimation method for network topology analysis, avoiding the arbitrariness of threshold selection in traditional network analysis. Globally, participants with IA presented higher MaxDeg and K, and lower Ecc, R, and Diam in the upper alpha band, and higher MaxDeg, MaxBC, and K, and lower R and Diam in the beta band. A previous study suggested that more random networks showed low clustering and a short path length, corresponding to MST's shorter diameters and higher leaf numbers (star‐like topology), while regular networks corresponded to the line‐like topology (Boersma et al., 2013). Our results indicate the MST brain network of HC participants tended to be line‐like, while the brain network of IA participants tended to be star‐like. These results imply that the brain of IA participants developed for randomization. MaxDeg, MaxBC, and K are all indexes showing the existence of high‐degree nodes or hubs (Anjomshoa et al., 2016). These indexes’ values were higher in the IA group, suggesting some brain regions have a greater cognitive burden than comparable regions, which may ultimately be a risk factor for crucial node overloaded. This phenomenon can also be inferred from the difference in Th properties, though no statistical significance was detected. From Table 2, we observed the Th value of the IA group was higher than the HC group. The Th of a line‐like and a star‐like topology approaches 0 and 0.5, respectively. For leaf numbers between these 2 extreme situations, Th can have higher values, such topology may reflect more optimal network organization that provide a tradeoff between node‐overload and efficient communication (Boersma et al., 2013; Fraga et al., 2016). In addition, Diameter and Ecc, metrics of network efficiency, correspond to path length in traditional network analysis. In a network with lower distance, information is efficiently processed between remote brain regions (Janssen et al., 2017).

In essence, the changes in the network measures mentioned above all point to the same phenomenon: the topological organization of college students with IA shift toward a more centralized, star‐like and random network compared to HC participants. IA as a behavioral addiction that was considered to share similar neurobiological abnormalities with substance addiction (Ding et al., 2013). Using graph theoretical analysis, studies revealed the brain network in heroin‐dependent individuals and young smokers may shift towards a random network (Zhang et al., 2016, 2017). However, we are aware that the results seem to deviate from other network studies in IA that have indicated a more regular network organization (Zhai et al., 2017) or unchanged topology (Hong, Zalesky et al., 2013; Lee et al., 2017; Wee et al., 2014). The reasons leading to this difference may be the ones mentioned in Section 4.1. Furthermore, previous studies have explored topological networks of IA from structural (Lee et al., 2017; Zhai et al., 2017) and functional aspects (Hong, Zalesky et al., 2013; Wee et al., 2014), and the applied graph theory methods were different. MST, used in our study, is more robust for estimation of network topology, while group differences obtained with conventional network analyses can go in any direction, depending on the choices made during the analysis (Tijms et al., 2013). In our study, participants with IA with lower diameter and a trend of increased leaf may indicate an alteration in the normal balance of network function.

In addition, the results of PLI connectivity and MST analysis of global network organization were inconsistent in different frequencies. The difference in PLI connectivity was mainly found in lower frequencies, while global MST differences were seen in higher frequencies. Since no other EEG study on IA has explored topological network‐revealed frequency effects, we can only speculate about this frequency significance. One might hypothesize that changes in different frequency bands reflected different aspects of a compensatory mechanism (van Diessen et al., 2016). Another reason may be that connectivity analysis and network analysis are two measures to explore the alteration of brain mechanism from different aspects (Fraga et al., 2016; Stam & van Straaten, 2012).

### 4.3 Alterations of hub location and regional MST measures

The occipital lobe is the visual processing center of the mammalian brain, containing most of the anatomical region of the visual cortex. It is thought to be responsible for visual function (Kojima & Suzuki, 2010). The occipital brain area plays a key role in visual processes and is involved in IA (Dong, Jie, & Du, 2012; Ling, Yue, Wenjie, & Fan, 2015). Internet game tasks can activate the vision center which is composed of the occipital gyrus (Du et al., 2011; Liu et al., 2016). IA participants showed decreased regional homogeneity in temporal, occipital, and parietal brain regions. These regions are thought responsible for visual and auditory functions (Dong et al., 2012). Our results show that in the lower frequency band, the most important node (as indicated by the highest degree and BC values) was located in the occipital brain region in HCs and, with increasing IA severity, became relatively less important. We speculate the reason for the reduced importance of the occipital lobe in IA participants was that internet users have long indulged in the internet need to pay full attention to each tiny change in the screen. Long‐term hypertension of visual attention can impair subjects’ visual functions (Dong et al., 2012). Therefore, the occipital lobe, which is very important in the HC group, is less important in the IA group, maybe because long‐term internet use has weakened their visual ability.

However, the importance of central and parietooccipital regions increased in IA compared with HC in our study, as shown by increased regional degree and BC values. We could consider this phenomenon a compensatory mechanism (Scheller, Minkova, Leitner, & Klöppel, 2014). In the case of HC subjects, this mechanism would not be needed. The occipital area was weakened in the brain network in IA participants, while the central and parietooccipital regions compensated for this malfunction. It also may reflect that some brain areas lose control within the network while others function in a more aberrant way (López et al., 2017), which would be in accordance with the activity‐dependent degeneration theory (Engels et al., 2015).

In addition, we took into consideration the social and emotional component of IA to explain the changed brain functions. IA may affect an individual's self‐control and understanding of their own and others' emotions, so internet addicts usually have negative emotions, such as impulsivity, low self‐esteem, depression, anxiety, loneliness, and even suicidal ideation (Sami, Danielle, Lihi, & Elena, 2018; Yücens & Üzer, 2018). These negative psychosocial factors may also affect the IA process. For example, Ding et al. (2014) suggested that impaired function of the prefrontal cortex may relate to high impulsivity in adolescents with IA, which may contribute directly to the IA process. Dieter et al. (2017) revealed decreased left middle and superior temporal gyrus activation while experiencing socially anxious words in internet gaming addicts. Additionally, risk taking is related importantly to addictive behaviors (Panwar et al., 2014). IA is regarded as behavioral addiction, and the lesser activation in the parietal neural processes underlying decision‐making has been systematically explored (Liu et al., 2017). Our data further suggest the parietal (P7) is a region of the brain that plays an important role in IA from resting‐state perspective. In general, the social and emotional components of IA which may affect the functions of the brain regions.

### 4.4 Correlation between global MST and IA severity

Measures of global MST were correlated with IA severity: more severe IA was correlated with higher MaxDeg and K, and with lower Ecc and Diam. Correlation results can be used as an auxiliary description of the relationship between behavior and brain topology change. Subjects with more severe IA tended to have a more random brain network. However, the results of correlation analysis are not as accurate as causal analysis. Future studies could increase the reliability of this trend by dividing several subgroups based on the degree of IA or conducting longitudinal research.

### 4.5 Strengths and Limitations

This study applied MST to IA analysis to explore the brain mechanism alterations related to IA for the first time. MST, containing the strongest connection in the original network, offers a nonarbitrary method for comparing networks. Thus, it allows us to better detect subtle network alterations. One strength is the use of PLI to measure FC, since it reduces the bias due to volume conduction and activity from common sources (Stam et al., 2007). Another strong point is that when we compared the differences of global PLI and MST measures between groups, age, sex and the power of each frequency band were taken into consideration as covariates (Vecchio et al., 2017).

Despite these strengths, there were several limitations to our study. First, although PLI is not affected by the volume conductor effect and is widely used in detecting real changes in functional networks (Fraga et al., 2016; van Dellen et al., 2014; van Diessen et al., 2016), the PLI may underestimate the FC because all zero‐lag (mostly short distance) networks are discarded in this measure, and it might be biased toward long‐distance connectivity (Engels et al., 2015). Second, conclusions about regional effects should be taken with caution due to the lower spatial resolution of EEG. Future studies should be combined with source location techniques to obtain comparable results to fMRI. Finally, some significant differences described in this study were not corrected for multiple testing. After Bonferroni or false discovery rate correction, there were no significant differences between groups. Therefore, these results are presented as an exploratory study that can be used as a guide for regions and measures that show a trend toward significance between IA and controls.

## 5 CONCLUSIONS

Our results revealed FC and topological differences between the IA and HC groups. We found evidence for increased FC and a more random organization in IA participants compared to HCs, and a decrease of relative functional importance of the visual processing area in IA participants. Together, these alterations can help us understand the influence of IA to brain mechanism. In addition, this study contributes to the literature by using MST to detect the neural differences between the groups and provides evidence that MST analysis is more sensitive in brain network analysis than traditional graph theory.

## CONFLICT OF INTEREST

We declare that we have no conflict of interest.

## AUTHOR CONTRIBUTIONS

HW and YS conceived and initiated the study in this paper. SB conducted the experiment. HW analyzed the data. HW and YS interpreted the results and drafted the manuscript. JL checked the reference. All authors reviewed the manuscript and approved the final version for the publication.
