# Supplementary material for: Random topology organization and decreased visual processing of internet addiction: Evidence from a minimum spanning tree analysis
Source: Brain Behav. 2019 Jan 31;9(3):e01218. doi: 10.1002/brb3.1218 (PMC6422800; doi:10.1002/brb3.1218)
Supplement: Supplementary file 1 [file BRB3-9-e01218-s001.docx]

**Table S1**

The results of global PLI and MST measures in theta, alpha1 and gamma frequency bands

|  | | IA(N=30) | | HC(N=30) | | *F* | *P* | *η^2^* |
| --- | --- | --- | --- | --- | --- | --- | --- | --- |
|  |  | M | SD | M | SD |  |  |  |
| theta | PLI | 0.069 | 0.056 | 0.057 | 0.048 | 0.753 | n.s. | 0.002 |
|  | Max Degree | 0.727 | 0.270 | 0.721 | 0.243 | 0.010 | n.s. | 0.000 |
|  | Eccentricity | 0.081 | 0.050 | 0.080 | 0.042 | 0.041 | n.s. | 0.000 |
|  | Max BC | 0.892 | 0.128 | 0.895 | 0.115 | 0.032 | n.s. | 0.000 |
|  | Kappa | 20.854 | 10.150 | 20.212 | 9.349 | 0.084 | n.s. | 0.000 |
|  | R | -0.767 | 0.209 | -0.660 | 0.271 | 2.892 | n.s. | 0.008 |
|  | Diameter | 0.095 | 0.066 | 0.094 | 0.056 | 0.015 | n.s. | 0.000 |
|  | Leaf | 0.922 | 0.095 | 0.923 | 0.078 | 0.011 | n.s. | 0.000 |
|  | Th | 0.523 | 0.062 | 0.521 | 0.055 | 0.032 | n.s. | 0.000 |
| alpha1 | PLI | 0.032 | 0.032 | 0.033 | 0.034 | 0.009 | n.s. | 0.000 |
|  | Max Degree | 0.858 | 0.196 | 0.801 | 0.214 | 1.179 | n.s. | 0.003 |
|  | Eccentricity | 0.062 | 0.037 | 0.073 | 0.038 | 1.353 | n.s. | 0.004 |
|  | Max BC | 0.963 | 0.075 | 0.929 | 0.101 | 1.933 | n.s. | 0.006 |
|  | Kappa | 25.503 | 7.733 | 22.999 | 8.863 | 1.566 | n.s. | 0.005 |
|  | R | -0.686 | 0.288 | -0.679 | 0.292 | 0.017 | n.s. | 0.000 |
|  | Diameter | 0.071 | 0.049 | 0.083 | 0.049 | 0.892 | n.s. | 0.003 |
|  | Leaf | 0.951 | 0.075 | 0.934 | 0.072 | 0.750 | n.s. | 0.002 |
|  | Th | 0.494 | 0.023 | 0.506 | 0.036 | 0.948 | n.s. | 0.003 |
| gamma | PLI | 0.040 | 0.036 | 0.039 | 0.050 | 0.002 | n.s. | 0.000 |
|  | Max Degree | 0.907 | 0.151 | 0.891 | 0.165 | 0.281 | n.s. | 0.001 |
|  | Eccentricity | 0.051 | 0.020 | 0.059 | 0.031 | 1.153 | n.s. | 0.003 |
|  | Max BC | 0.977 | 0.053 | 0.962 | 0.082 | 0.672 | n.s. | 0.002 |
|  | Kappa | 27.665 | 6.237 | 26.844 | 7.010 | 0.429 | n.s. | 0.001 |
|  | R | -0.786 | 0.266 | -0.757 | 0.270 | 0.425 | n.s. | 0.001 |
|  | Diameter | 0.055 | 0.023 | 0.065 | 0.038 | 0.902 | n.s. | 0.003 |
|  | Leaf | 0.976 | 0.024 | 0.963 | 0.051 | 0.572 | n.s. | 0.002 |
|  | Th | 0.501 | 0.025 | 0.503 | 0.032 | 0.145 | n.s. | 0.000 |

*Notes:* MST, minimum spanning tree; PLI, phase lag index; BC, betweenness centrality; R, degree correlation; Th, tree hierarchy.

n.s.= non-significant.

**Table S2.** The correlation between MST indices and IAT scores for the IA group and the HC group respectively

| **Bands** | **Measures** | **IAT** | |
| --- | --- | --- | --- |
|  |  | ***r_IA_ r_HC_*** | ***P_IA_ P_HC_*** |
| Alpha2 | MaxDeg | 0.078 0.215 | 0.681 0.254 |
|  | Ecc | -0.151 -0.080 | 0.425 0.674 |
|  | K | 0.084 0.193 | 0.659 0.306 |
|  | Diam | -0.161 -0.045 | 0.396 0.815 |
| Beta | MaxDeg | 0.178 0.068 | 0.348 0.719 |
|  | K | 0.162 0.055 | 0.392 0.772 |

*Notes:* MaxDeg, maximum degree; Ecc, eccentricity; K, kappa; Diam, diameter.


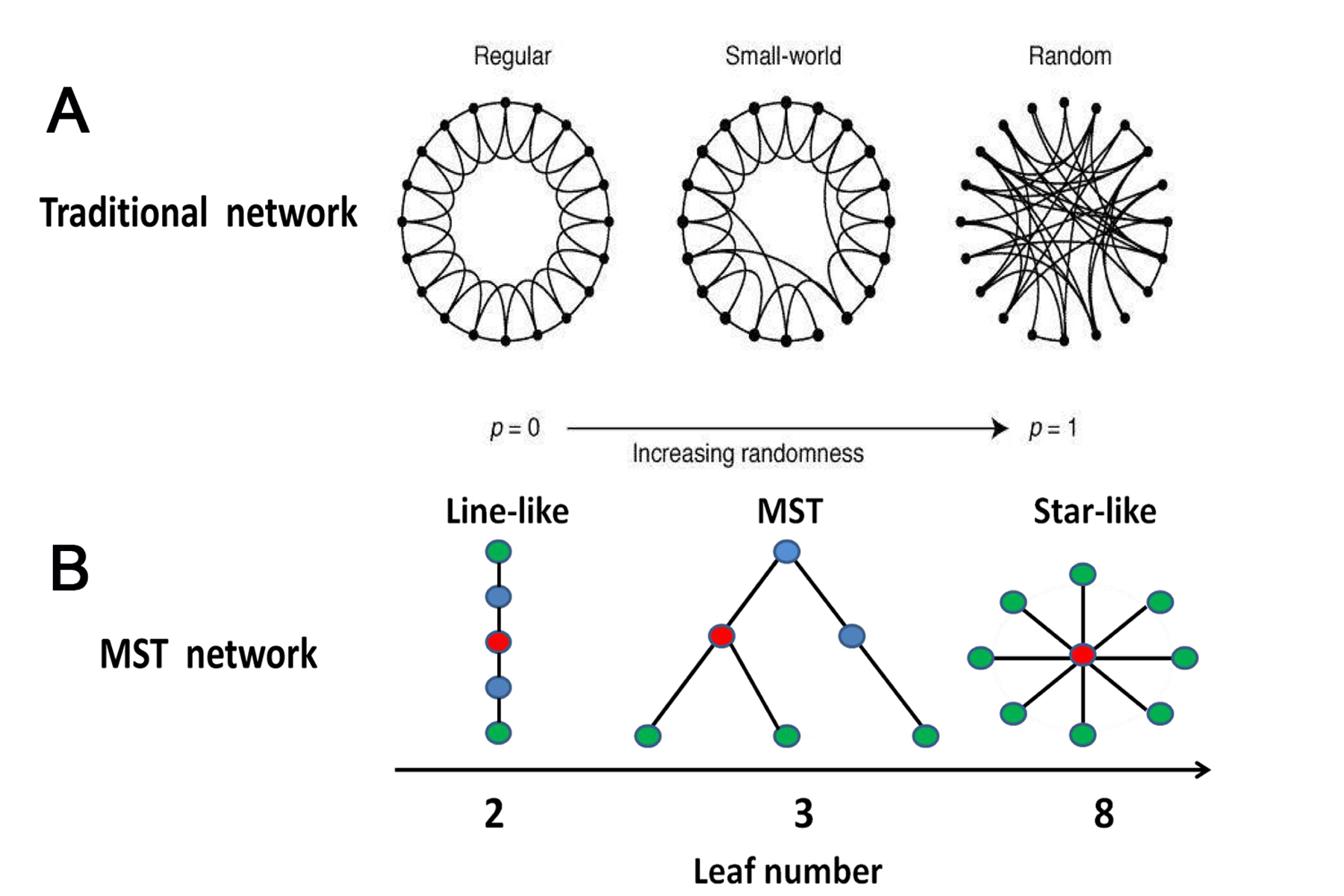


**Figure. S1.** Extreme topologies of traditional (A) and MST (B) networks. The line-like and star-like configurations might be translation of regular and random networks, respectively. In B, the red nodes indicated core nodes, green nodes indicated leaf nodes.


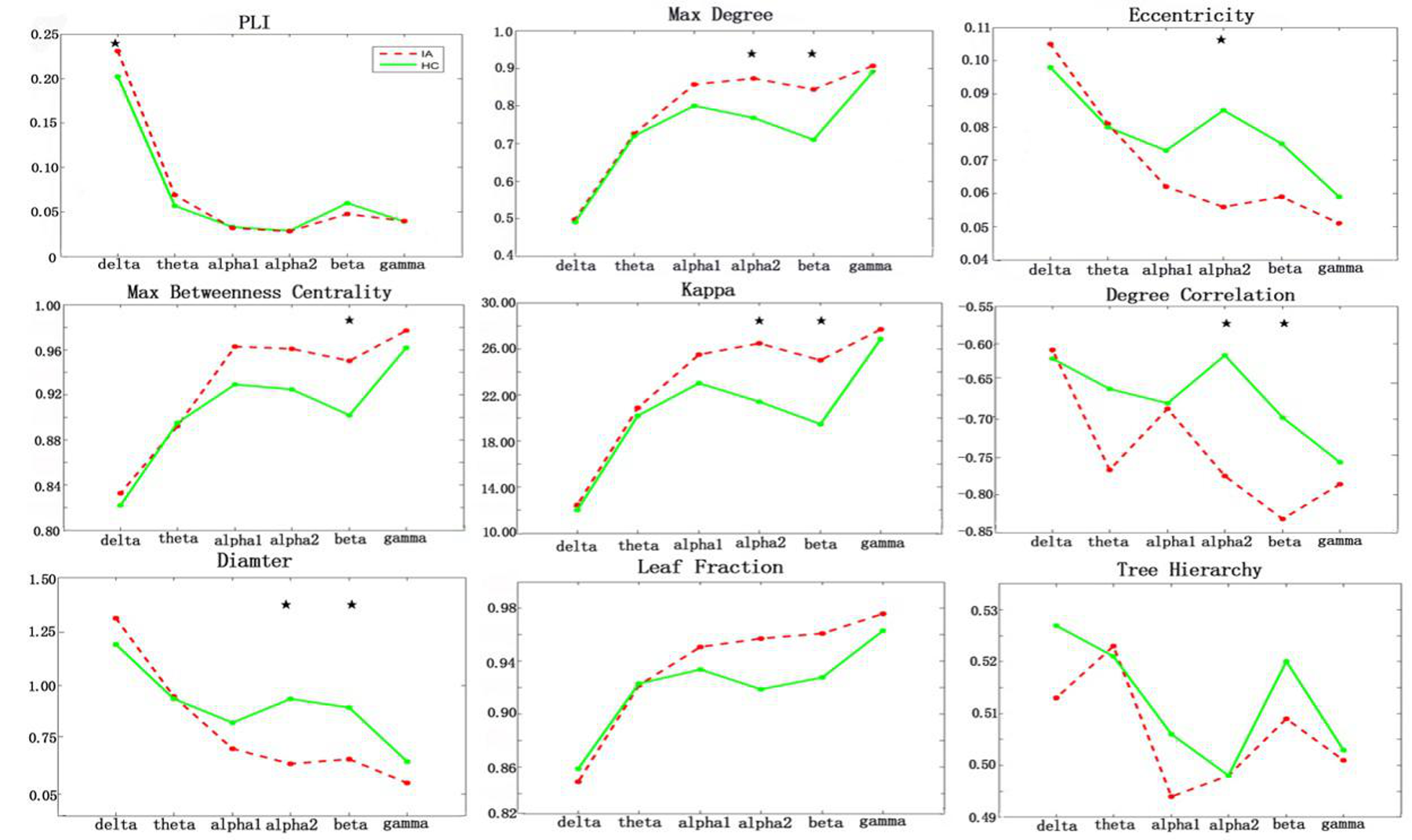


**Figure. S2.** Descriptive statistics of the MST metrics of the IA and HC networks for each frequency band. Red dotted line represented the IA group, green solid line represented the HC ones. And the asterisk (★) indicated the significant differences between groups were less than 0.05.
